# Impact of Socioeconomic Inequality on Access, Adherence, and Outcomes of Antiretroviral Treatment Services for People Living with HIV/AIDS in Vietnam

**DOI:** 10.1371/journal.pone.0168687

**Published:** 2016-12-22

**Authors:** Bach Xuan Tran, Jongnam Hwang, Long Hoang Nguyen, Anh Tuan Nguyen, Noah Reed Knowlton Latkin, Ngoc Kim Tran, Vu Thi Minh Thuc, Huong Lan Thi Nguyen, Huong Thu Thi Phan, Huong Thi Le, Tho Dinh Tran, Carl A. Latkin

**Affiliations:** 1 Institute for Preventive Medicine and Public Health, Hanoi Medical University, Hanoi, Vietnam; 2 Johns Hopkins Bloomberg School of Public Health, Baltimore, Maryland, United States of America; 3 Department of Health Promotion, Daegu University, Gyeongsan, Republic of Korea; 4 School of Medicine and Pharmacy, Vietnam National University, Hanoi, Vietnam; 5 Institute for Global Health Innovations, Duy Tan University, Da Nang, Vietnam; 6 Woolcock Medical Research Institute, Hanoi, Vietnam; 7 Department of Immunology and Allergy, National Otolaryngology Hospital, Hanoi, Vietnam; 8 Authority of HIV/AIDS Control, Ministry of Health, Hanoi, Vietnam; 9 Department of Hepatobiliary Surgery, Vietnam-Germany Hospital, Hanoi, Vietnam; Simon Fraser University, CANADA

## Abstract

**Background:**

Ensuring an equal benefit across different patient groups is necessary while scaling up free-of-charge antiretroviral treatment (ART) services. This study aimed to measure the disparity in access, adherence, and outcomes of ART in Vietnam and the effects of socioeconomic status (SES) characteristics on the levels of inequality.

**Methods:**

A cross-sectional study was conducted in 1133 PLWH in Vietnam. ART access, adherence, and treatment outcomes were self-reported using a structured questionnaire. Wealth-related inequality was calculated using a concentration index, and a decomposition analysis was used to determine the contribution of each SES variable to inequality in access, adherence, and outcomes of ART.

**Results:**

Based on SES, minor inequality was found in ART access and adherence while there was considerable inequality in ART outcomes. Poor people were more likely to start treatment early, while rich people had better adherence and overall treatment outcomes. Decomposition revealed that occupation and education played important roles in inequality in ART access, adherence, and treatment outcomes

**Conclusion:**

The findings suggested that health services should be integrated into the ART regimen. Furthermore, occupational orientation and training courses should be provided to reduce inequality in ART access, adherence, and treatment outcomes.

## Introduction

Globally, a rapid scale-up of antiretroviral therapy (ART) service has sustainably contributed to diminishing the burden of HIV epidemic in both individual and social perspectives [1–3]. By the end of 2015, over 17 million people living with HIV (PLWHs) worldwide were on ART, two million of whom initiated their treatment the same year [4]. ART helps PWLH to prolong virologic suppression, bolster their immune systems, decrease opportunistic infections, and therefore, lengthen their lifespan and improve their quality of life [5–9]. The patients have to strictly adhere to their regimes in order for the treatment to be successful and to prevent drug resistance [10]. The World Health Organization (WHO) recommends that PLWHs should start ART irrespective of their CD4 cell count [6].

In spite of the critical role of ART, there are widespread inequalities in accessing and utilizing this service [11–14]. International research suggests that lower socioeconomic status (SES) was closely related to delayed ART initiation, lower treatment adherence, and poorer outcomes [11–14]. For example, previous studies showed the positive association between education and early HIV diagnosis and ART initiation [11,15,16]. Furthermore, WHO emphasized in “Treatment 2.0” guideline that wealthier PLWH were more likely to have access to better HIV/AIDS care services [17]. Another study suggests that unemployed people were more likely to withdraw from treatment [10]. Since socioeconomically disadvantaged groups have been extremely vulnerable to the HIV epidemic, the impacts of SES on access, adherence and outcomes of antiretroviral treatment services among PLWH should be carefully elucidated.

In Vietnam, in the first half of 2016, there were about 227.225 people reported HIV/AIDS positive, a large portion belonging to some of the most at-risk populations such as people injecting drugs, men who have sex with men, and female sex workers [18]. Thanks to funding from international donors, there are currently more than four hundred outpatient clinics providing ART and other HIV-related services free-of-charge [19,20]. However, only 48% of PLWH have access to ART [18]. Furthermore, delayed ART access and utilization are still common in Vietnam. Several reports suggest that more than 50% of patients started treatment when their CD4 cell count was less than 100 cell/m^3^ [21,22]. Lower educational attainment, lower income, high out-of-pocket payments, and social stigma against PLWH are considered to be major contributors to the delayed use of ART in Vietnam [23]. The Vietnamese government has shown a strong commitment to addressing these issues by implementing national HIV/AIDS control strategies that 80% of PLWH will receive ART-related services by 2020 [24].

Despite an increasing concern in health-care inequalities topic in Vietnam [25], limited empirical studies show the relationship between SES and primary aspects of ARV treatment. A few studies showed that SES inequalities could be directly and indirectly associated with the risk of HIV infection [26] and the level of stigma against PLWH [27]. However, none of them mentions whether PLWH could have timely access to ART services, adhere to the regimen, and achieve successful treatment outcomes without depending on SES. Moreover, in the literature, most treatment outcomes are only measured in biological indicators, such as CD4 count or HIV viral load. These measures alone cannot take into account larger socioeconomic factors, which affect the overall success of and access to ART in different populations over an extended period of time. Therefor it is necessary to develop a specific indicator that can display the long-term effects of ART. This study aimed to measure the socioeconomic inequalities in access, adherence, and outcomes of ART services for Vietnamese PLWH; and identify contributors to those disparities.

## Materials and Methods

### Study settings and subjects

A cross-sectional study was performed from January to September 2013 in two leading HIV epicenters in northern Vietnam, theHanoi capital and Nam Dinh province, with 20,762 and 3,781 PLWH, respectively [28]. Eight ART clinics, five in Hanoi and three in Nam Dinh, were included in this study. Those clinics were selected based on following criteria: 1) including clinics in central-, provincial- and district-levels; 2) having an adequate number of PLWH currently on ART.

The participants must meet following criteria: 1) age ≥ 18 years old; 2) currently being on ART; 3) agreeing to participate in the study and providing written informed consent; 4) having the physical and psychological capacity to answer questionnaire within 25–30 minutes. A convenience sampling technique was used to recruit patients. People who were visiting the clinics during the study period were invited to enroll in the study. A total of 1133 HIV/AIDS patients participated in this study.

### Data collection

The collection team included well-trained master students in public health and HIV/AIDS experts. A structure questionnaire was used, which included information about socio-demographic and clinical characteristics (i.e. HIV stage, history of drug use, initiated and current CD4 cell count, ART adherence and duration) as well as health outcomes (health-related quality of life—HRQOL).

### Variables

#### Socio-economic characteristics

We investigated some variables namely age, gender, religion, marriage status, educational attainment, occupations and self-reported household income in the last 12 months. In general practice, income, education, and occupation are frequently used to assess socioeconomic status (SES), each with their own advantages and disadvantages [29]. In this case, income was used to assess SES and classified the socio-economic status into five quintiles, with the 1st quintile representing the poorest and the 5^th^ quintile representing the richest.

#### ART access and adherence

Initiated CD4 cell count was retrieved from medical records to measure ART service access. Based on this indicator, we assessed whether people received late ART treatment or not. At the time when the study was conducted, the guidelines of the WHO recommended that PLWH should be treated when CD4 cell count reaches 350 [5]. This threshold was utilized to assess late ART access.

The ART adherence within 30 days of respondents was measured by using a 100-point visual analogue scale (VAS), with the value of 0 meaning complete non-adherence and the value of 100 suggesting complete adherence. [30]. The threshold of ≥ 95% was selected for optimal adherence [31].

#### ART outcome of treatment

Self-reports about the latest CD4 cell count and health-related quality of life were collected from patients. HRQOL was measured by using EQ-5D-5L instrument in the Vietnamese version, which was validated elsewhere [32]. This tool measures five dimensions including mobility, self-care, usual activities, pain/discomfort and anxiety/depression with five response levels: no problems, slight problems, moderate problems, severe problems, and extreme problems [33]. The combination of responses gives 3125 health states with weighting to have aggregate single index [32]. Furthermore, the EQ-VAS (visual analogue scale) measures the self-rated health on a 20-cm vertical scale, with the endpoint ranges from 0 to 100 point, labeled ‘the worst health you can imagine” and ‘the best health you can imagine” [33]. A prior study suggested that the VAS score of 80 was equal to the average score of normal Vietnamese population [9]. Therefore, this threshold was chosen for optimal HRQOL.

#### Concentration Index (CI) and decomposition of the CI

Concentration Index (CI) was applied to assess the income-related inequality in health outcomes and health care services. The CI is a popular tool to measure health and health care inequality in the field of health policy and health economics research [34]. It is obtained by quantifying the area between the concentration curve (which individuals, ranked by socioeconomic status, are plotted against the cumulative share of health outcome or health care service variable) and the line of equity (45-degree line, obtained from concentration curve).

In general, the CI ranges between -1 and +1, and CI is zero when there is no difference in sharing of health or health care outcome among different SES ranking groups. Positive CI value indicates that the outcome is concentrated in higher SES groups, which can be called “pro-rich”; and the opposite is true for negative values.

Decomposition of the CI method was applied to identify major contributors to the observed income-related inequality. The basic idea of decomposing the CI is quantifying each contributor to the observed CI, as the overall CI is a sum of the contribution from each factor and residual.

### Statistical analysis

Data was analyzed using STATA version 12 (Stata Corp. LP, College Station, United States of America). Descriptive analysis was used for demographic characteristics of respondents as well as HRQOL, adherence to treatment and changes in CD4 level. The disparity in access, adherence, and outcome of treatment between people with different income per capita regarding quintiles was determined using CI.

### Ethics approval

The protocol of this study was reviewed and approved by the Vietnam Authority of HIV/AIDS Control's Scientific Research Committee. Written informed consent was obtained from all participants. Patients could withdraw at any time without it influencing their current treatment.

## Results

**Table 1**describes the demographic characteristics of respondents. A total of 1133 patients enrolled in this survey (90% response rate). Most of them were male (58.7%) between 30 and 45 years old (75.3%), living with their spouses or partners (61.2%) and having at least high-school education (41.2%). The majority of respondents were self-employed (41.4%) and workers/farmers (24.9%).

**Table 1:** Demographic characteristics of respondents.

Characteristics	Wealth status								p-value
	≤ Poor		Middle		≥ Rich		Total		
	n	%	n	%	n	%	n	%	
**Level of clinic**									
Central	86	18.4	66	26.5	125	30.1	277	24.5	<0.01
Province	51	10.9	24	9.6	24	5.8	99	8.7	
District	331	70.7	159	63.9	267	64.2	757	66.8	
**Gender**									
Female	187	40.0	108	43.4	173	41.6	468	41.3	0.67
**Age**									
18–<25	9	1.92	4	1.61	10	2.4	23	2.0	0.04
25–<30	52	11.11	32	12.85	65	15.6	149	13.2	
30–<35	170	36.32	83	33.33	160	38.5	413	36.5	
35–<40	121	25.85	68	27.31	119	28.6	308	27.2	
40–<45	66	14.1	31	12.45	35	8.4	132	11.7	
> = 45	50	10.68	31	12.45	27	6.5	108	9.5	
**Marital status**									
Single	65	13.9	30	12.1	74	17.8	169	14.9	0.20
Live with spouse/partner	285	60.9	164	65.9	244	58.7	693	61.2	
Divorced/ Widow	118	25.2	55	22.1	98	23.6	271	23.9	
**Educational attainment**									
≤ Second	340	72.8	139	55.8	170	40.9	649	57.3	<0.01
High	114	24.4	87	34.9	161	38.7	362	32.0	
Vocational training	5	1.1	12	4.8	37	8.9	54	4.8	
University	8	1.7	11	4.4	48	11.5	67	5.9	
**Employment**									
Unemployed	132	28.2	51	20.5	49	11.8	232	20.5	<0.01
Self-employed	131	28.0	123	49.4	215	51.7	469	41.4	
Officers	8	1.7	15	6.0	57	13.7	80	7.1	
Workers, Farmers	176	37.6	47	18.9	59	14.2	282	24.9	
Other jobs	21	4.5	13	5.2	36	8.7	70	6.2	
**Religion**									
Cult of ancestors	404	86.3	222	89.2	375	90.1	1001	88.4	0.19
Others	64	13.7	27	10.8	41	9.9	132	11.7	
**History of drug use**									
Yes	178	38.03	86	34.54	138	33.17	402	35.48	0.30

The ART access, adherence, and outcomes of respondents are depicted in **Table 2.** The majority of them received treatment for more than two years (94.7%). Approximately half of the patients were at asymptomatic (41.6%), while 16% reported symptoms and 10.8% reported to be at the disease stage of AIDS. However, over one-third of the patients did not know their HIV disease stages. Most of the respondents had low CD4 cell counts (<200 cells) at ART initiation (65.6%). However, the percentage of people with low CD4 cell counts was remarkably higher in the poor group than the rich group (p<0.05). About half of the patients had current CD4 cell counts at 350 cells or higher (47.1%) and only 25.7% of patients reported to have optimally adhered to ARV treatment during the last 30 days (VAS ≥ 95%).

**Table 2:** ART access, adherence and outcome of respondents regarding to income quintiles.

Characteristics	≤ Poor		Middle		≥ Rich		p-value
	n	%	n	%	n	%	
**Duration of ART**							
< 2 years	21	4.5	7	2.8	22	5.3	0.07
2–< 4 years	140	29.9	72	28.9	154	37.0	
4–< 7 years	196	41.9	104	41.8	140	33.7	
≥ 7 years	111	23.7	66	26.5	100	24.0	
**HIV stage**							
Asymptomatic	182	38.9	101	40.6	173	41.6	0.36
Symptomatic	78	16.7	48	19.3	67	16.1	
AIDS	35	7.5	21	8.4	45	10.8	
Unknown	173	37.0	79	31.7	131	31.5	
**Initiated CD4 cell count**							
<100	274	58.6	147	59.0	199	47.8	0.02
100- < 200	75	16.0	37	14.9	70	16.8	
200- < 350	83	17.7	43	17.3	99	23.8	
> = 350	36	7.7	22	8.8	48	11.5	
**Current CD4 cell counts**							
<100	108	23.1	53	21.3	70	16.8	0.25
100–200	51	10.9	21	8.4	43	10.3	
200–350	120	25.6	62	24.9	107	25.7	
> = 350	189	40.4	113	45.4	196	47.1	
**ART adherence (VAS ≥ 95%)**							
Yes	146	31.2	60	24.1	107	25.7	0.07
No	322	68.8	189	75.9	309	74.3	

The results of **Table 3**suggest that participants with greater wealth had better HRQOL. The proportion of people who had problems in all of five of the characteristics of mobility, self-care, usual activities, pain/discomfort and anxiety/depression decreased from poor to rich quintiles (p < 0.05). The overall EQ-5D score and EQ-VAS were also significantly higher in the rich group than the poor group (p<0.05). The proportion of optimal HRQOL people in the rich group was double those in the poor group (p<0.05).

**Table 3:** HRQOL of respondents by household’s economic status.

Characteristics	≤ Poor		Middle		≥ Rich		p-value
	n	%	n	%	n	%	
**Mobility**							
Have problems	124	26.5	53	21.3	55	13.2	<0.01
No problems	344	73.5	196	78.7	361	86.8	
**Self-care**							
Have problems	64	13.7	20	8.0	26	6.3	<0.01
No problems	404	86.3	229	92.0	390	93.8	
**Usual activities**							
Have problems	103	22.0	41	16.5	44	10.6	<0.01
No problems	365	78.0	208	83.5	372	89.4	
**Pain/discomfort**							
Have problems	203	43.4	100	40.2	124	29.8	<0.01
No problems	265	56.6	149	59.8	292	70.2	
**Anxiety/depression**							
Have problems	250	53.4	121	48.6	138	33.2	<0.01
No problems	218	46.6	128	51.4	278	66.8	
**Optimal HRQOL**							
**≥** 80 VAS score	117	25.0	99	39.8	210	50.5	<0.01
< 80 VAS Score	351	75.0	150	60.2	206	49.5	
	**Mean**	**SD**	**Mean**	**SD**	**Mean**	**SD**	
EQ-5D index	0.74	0.26	0.78	0.22	0.84	0.21	<0.01
VAS	63.88	17.42	69.36	17.31	74.09	15.40	<0.01

**Fig 1:**
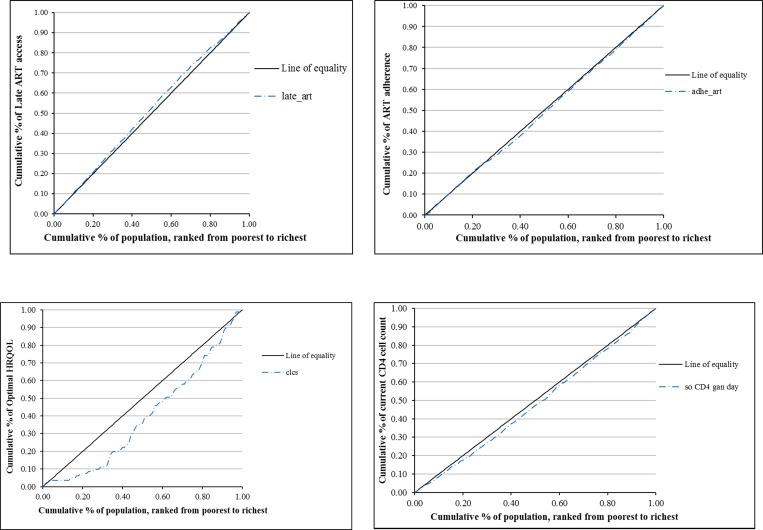
Concentration curves for the severity of inequality on access, adherence, and outcomes of ART services. (A) Late ART access (B) ART adherence treatment (C) HRQOL (D) Current CD4 cell count.

The severity of inequality was shown in **Fig 1.** A slight inequality in timely access to ART was found with CI = -0.027, showing that timely ART access was concentrated among poor people. In contrast, the CI for treatment had a positive value (CI = 0.021), indicating poor people experienced greater difficulty in ART adherence. In term of treatment outcomes such as HRQOL and current CD4 cell count, the CI was also a positive value (CI = 0.196 and 0.051, respectively), revealing an inequality in outcomes that favored the rich.

The results of a decomposition analysis are shown in **Table 4.** Education and age were two main factors contributing to inequality in access to ART (23% and 18%, respectively). These two factors were also the main contributors to the disparity in treatment outcomes based on CD4 count measurements, where occupation contributed 24%, and age contributed 15% to the CI. Meanwhile, the inequality in treatment adherence was mainly attributed to education and occupation (47% and 16%, respectively). The negative value of the contribution of occupation to adherence inequality showed that while the overall inequality is pro-rich, occupation had a reverse effect, lessening the disparity. Education and occupation were also the main contributors to the inequality in treatment outcomes when the quality of life is measured. Notably, a large part of all four indexes is explained by the error term of the regression.

**Table 4:** Decomposition of SES inequalities in ART access, adherence and outcome treatments.

Characteristics	Late access to ART		ART adherence		HRQOL		Current CD4 cell count	
	Marginal effect	Contribution (%)	Marginal effect	Contribution (%)	Marginal effect	Contribution (%)	Marginal effect	Contribution (%)
**Gender**	0.000	0%	0.000	0%	0.000	0%	0.000	0%
**Age**	-0.005	18%	0.000	0%	0.012	6%	0.004	15%
**Marital status**	0.000	2%	0.001	4%	-0.001	0%	0.000	2%
**Education**	-0.006	23%	0.010	47%	0.016	8%	0.001	4%
**Occupation**	-0.001	2%	-0.003	-16%	0.032	16%	0.006	24%
**Religion**	0.001	-2%	-0.001	-4%	0.001	0%	0.000	1%
**Level of clinic**	-0.001	2%	0.000	1%	0.011	5%	0.000	-2%
**Drug use**	-0.001	4%	0.001	4%	0.000	0%	0.000	1%
**HIV stage**	0.001	-5%	0.000	0%	-0.003	-2%	-0.002	-6%
**Duration of ART**	-0.001	5%	0.000	0%	-0.001	-1%	-0.001	-5%
**Residual**	-0.014	51%	0.013	62%	0.131	67%	0.017	68%
**Inequality (total)**	-0.027		0.021		0.196		0.025	

## Discussion

This study used a concentration index (CI) to investigate the presence of inequality in ART access, adherence and treatment outcomes among PLWH in Vietnam. We also decomposed the CI to identify the contributors of some SES variables to the inequalities. Our analysis indicated that the poor tended to have more timely access ARV treatment but had lower HRQOL, as compared to the rich. Furthermore, decomposition results suggested the minor contributions of socioeconomic factors to these inequalities.

Timely access to ART service plays a crucial role in HIV treatment, as delaying ART treatment can lead to an increased risk of HIV-related opportunistic infections and mortality, poor responses to treatment, and reduced life expectancy [35–37]. This study showed that the majority of respondents had late ART initiation, which was consistent with previous reports in Vietnam and other developing countries [21,23,38]. Nonetheless, we found a small pro-poor pattern in ART initiation regarding different SES groups (CI = -0.027). This value of CI is considerably small compared to the inequality in accessing general health care service in Vietnam (CI = 0.10–0.26) [39]. A possible explanation is that since ART has been provided freely in Vietnam, people can access treatment regardless of socioeconomic disadvantages. However, as foreign funding will decrease rapidly in the upcoming years, PLWH will have to pay for their own medications. This suggests that the income-related inequality will be worse if subsidies are unavailable for poor PLWH, who cannot afford ART treatment [40].

Compliance to ART treatment is a strict requirement for high effectiveness in HIV treatment [5,41]. Overall, the results suggest that most of the patients had good adherence to ART treatment. The results also indicated that the relationship between SES and inequality in ART adherence was relatively small among PLWH. This finding is similar to other prior studies, which indicated that socioeconomic factors might not have a significant impact on adherence among patients [42,43].

The disparities in treatment outcome varied based on CD4 cell counts and quality of life measurements. The positive value of CI represented a pro-rich tendency in the treatment outcomes. However, low concentration indexes when using CD4 cell counts indicated that the poor and the rich experienced somewhat similar clinical effectiveness of ART. This result was consistent with a previous longitudinal study, which found that SES did not have a significant influence on the change in CD4 cell count [44]. When quality of life measurement was utilized, the result showed a greater level of inequality; in particular, people with better SES tended to have better HRQOL. This can be attributed to the fact that people with high SES have a higher chance to access better living conditions and good health care services than poor people. This finding was consistent with prior studies in Vietnam that lower income, unemployment, and lower education attainment were related to lower HRQOL [32,45].

In addition, the difference can be explained by the distinct nature of each indicator. CD4 cell count is purely a clinical indicator, which is mainly affected by treatment regimen, drug quality, and the patient’s adherence to treatment. In the context of Vietnam, ART was received free of charge with comparable quality in the whole country, and with a similar level of adherence, the CD4 cell count does not significantly vary. Meanwhile, EQ-5D-5L scores and VAS scores reflected the psychological aspect of health, as well as the patients’ outlook towards living with HIV, which is mainly affected by individual socioeconomic status. A prior study about the psychometrics of the EQ-5D-5L instrument suggested that this tool has the potential to contribute to HIV treatment monitoring, along with clinical indicators such as CD4 cells and viral load [32]. Notably, in this study, VAS score for HRQOL was used to identify the optimal threshold instead of EQ-5D-5L index. According to a recent meta-analysis, VAS demonstrated good reliability and better discriminate validity than EQ-5D-5L in HIV treatment monitoring [8,32]. Additionally, EQ-5D-5L, as well as other generic instruments such as EQ-5D-3L, can have a high ceiling effect, which can influence the responsiveness of patients [8].

Decomposition revealed the contribution of various SES characteristics to the level of inequality, which is mostly affected by education and occupation. The results also showed that inequality in access, adherence, and outcomes of ARV treatment had patterns similar to the contributions of SES characteristics in comparison to those of other chronic diseases [46–48]. Meanwhile, high residual errors meant that a large proportion of contributions to inequalities were unexplained factors. For example, a study indicated that people experiencing discrimination and culture barriers were more likely to withdraw from treatment [49]. In addition, low quality of ART services was a factor that reduced the HRQOL of ART patients [45]. Further studies are needed to elucidate the impacts of a range of factors such on the levels of the individual (e.g. stigma), community (e.g. culture) and facility (e.g. quality of services, distance to access, etc.).

Although PLWH could access to ART without income barriers, the proportion of people receiving late ARV treatment was high. It suggested that HIV-related services should be effectively and efficiently scaled-up, as well as integrated into other health care facilities to improve their accessibility [23]. Integrating ART into other health care services will narrow the distance gap to access to this service, as well as reduce the operational cost of ART clinics, and promote community engagement to support the treatment [17,50]. A review of Suthar et al. (2014) suggests that integrating ART services with other services such as maternal care, tuberculosis, and drug addiction treatment enhanced ART coverage significantly [51].

Given the the dramatic contribution of occupational factors to the inequalities, vocational training and job referrals should be considered as potential interventions along with providing ART services. These have been shown to be effecting in improving HRQOL in Vietnam [9]. Moreover, financial mechanisms such as national subsidies or health insurance schemes should be provided, as theyare potentially beneficial for PLWH in reducing income-related inequalities in the access and utilization of ART services.

Third, in addition to the clinical measurements of outcomes such as CD4 cell count and viral load, HRQOL, as well as the capacity for daily living should be used as regular monitoring tools in HIV treatments, especially among poor people. For clinical practice, a meta-analysis of Bach et al. (year) suggested that during the course of ART treatment, HRQOL measurements were sensitive and responsive to the long-term changes of clinical experience among PLWH, which can help physicians to provide timely support to the patients [8]. For research purposes, longitudinal information from those measurements could serve as a vital indicator for economic and clinical evaluation in the field of HIV/AIDS [8].

To our knowledge, this is the first study that evaluated the socio-economic disparities in ART access, adherence, and outcomes of treatment among PLWH in the context of the concentrated epidemic of HIV in Vietnam. However, several limitations should be taken into consideration. First, the information collected was mainly based on patient self-reporting, which can lead to recall bias or over-/under-estimate the true value of income or HRQOL. To minimize this bias, we used different questions to recheck the information to ensure the reliability of the answers. Second, although viral load is generally more accurate than CD4 cell counts in measuring the effects of ART, it was not utilized in this study. Third, the convenient sampling technique limits the accuracy of the generalization of the entire population. Furthermore, the study utilized cross-sectional design, which does not allow for conclusions regarding the causal relationships among factors. A longitudinal study should be conducted to monitor and measure ART access, adherence, and outcomes of treatment regarding inequity in order to provide more reliable evidence for policy-making. In addition, the SES was measured by income per-capital alone, which lacks a more comprehensive view of the respondents’ SES.

## Conclusions

In conclusion, this study revealed the slightly pro-poor inequality in timely ART access, but pro-rich disparities in ARV treatment outcomes, especially in terms of HRQOL measurements. The findings also showed the minor contributions of socio-economic factors to inequality in ART access, adherence, and treatment outcomes among PLWH. These results can contribute to the development of policies geared toward greater equity in HIV care in Vietnam.
